# Fabrication of Electrospun Porous TiO_2_ Dielectric Film in a Ti–TiO_2_–Si Heterostructure for Metal–Insulator–Semiconductor Capacitors

**DOI:** 10.3390/mi15101231

**Published:** 2024-09-30

**Authors:** Jin-Uk Yoo, Tae-Min Choi, Sung-Gyu Pyo

**Affiliations:** School of Integrative Engineering, Chung-Ang University, 84, Heukseok-ro, Dongjak-gu, Seoul 06974, Republic of Korea; wlsdnr5771@naver.com (J.-U.Y.); c79411@gmail.com (T.-M.C.)

**Keywords:** MIS, electrospinning, TiO_2_, leakage current, electrospun dielectric layer

## Abstract

The development of metal–insulator–semiconductor (MIS) capacitors requires device miniaturization and excellent electrical properties. Traditional SiO_2_ gate dielectrics have reached their physical limits. In this context, high-k materials such as TiO_2_ are emerging as promising alternatives to SiO_2_. However, the deposition of dielectric layers in MIS capacitors typically requires high-vacuum equipment and challenging processing conditions. Therefore, in this study, we present a new method to effectively fabricate a poly(vinylidene fluoride) (PVDF)-based TiO_2_ dielectric layer via electrospinning. Nano-microscale layers were formed via electrospinning by applying a high voltage to a polymer solution, and electrical properties were analyzed as a function of the TiO_2_ crystalline phase and residual amount of PVDF at different annealing temperatures. Improved electrical properties were observed with increasing TiO_2_ anatase content, and the residual amount of PVDF decreased with increasing annealing temperature. The sample annealed at 600 °C showed a lower leakage current than those annealed at 300 and 450 °C, with a leakage current density of 7.5 × 10^−13^ A/cm^2^ when Vg = 0 V. Thus, electrospinning-based coating is a cost-effective method to fabricate dielectric thin films. Further studies will show that it is flexible and dielectric tunable, thus contributing to improve the performance of next-generation electronic devices.

## 1. Introduction

In recent decades, the advancement of memory device structures, particularly in gate and capacitor dielectrics, has driven significant innovation. As device dimensions continue to scale down, high-k dielectric materials, such as titanium dioxide (TiO_2_) [1,2], hafnium dioxide (HfO_2_) [3], and zirconium dioxide (ZrO_2_) [4], have been increasingly integrated into cutting-edge technologies like FinFETs and high-k metal gate (HKMG) structures to mitigate leakage current and short-channel effects [5].

Among these materials, TiO_2_ stands out due to its exceptionally high dielectric constant, ranging between 80 and 120, depending on whether it crystallizes in the anatase or rutile phase [6]. However, despite its high dielectric constant, TiO_2_ struggles with controlling leakage currents, primarily due to its relatively narrow band offset. To enhance its electrical properties, reducing oxygen vacancies and improving structural order through annealing processes are critical, as the phase transformation from anatase to rutile significantly impacts both its dielectric behavior and leakage characteristics [7,8,9,10].

While these high-k materials deliver superior performance, they have deposition methods such as physical vapor deposition (PVD) [11], chemical vapor deposition (CVD) [12], plasma-enhanced CVD (PECVD) [13], and atomic layer deposition (ALD) [10,14,15,16]. However, these deposition methods involve long processing times and high costs, owing to the use of ultrahigh vacuum systems, and they are limited by equipment space requirements [17]. Therefore, it is crucial to identify novel techniques that can significantly reduce the deposition time and cost, while also enabling deposition at the nano- and microscale under room temperature and atmospheric pressure conditions.

In the electrospinning process, nanoscale fibers are fabricated by applying a high voltage to a polymer solution [18,19]. In general, electrospun one-dimensional fibers are formed when the solution concentration is above the critical point; however, when the concentration is below the critical point, a porous film ranging from tens of nanometers to hundreds of micrometers can be formed by changing from electrospinning to electrospraying. In addition, depending on the metal nozzle type, it is possible to produce fibers with hollow, core–shell, and porous structures. Also, the deposition method using electrospinning does not use vacuum equipment and the equipment itself is inexpensive, so the process cost difference is about 5–10 times compared to the conventional method. The advantages of electrospinning are not the only ones. The film adhesion can be adjusted according to the type of organic material, and the dielectric constant can be controlled by tuning the doping material; hence, this approach can be developed into a coating technology with unlimited potential [20,21,22,23,24].

In this paper, an anatase TiO_2_ dielectric layer was deposited on a p-type (100)-oriented bare wafer using a simple, fast, and low-cost PVDF-based electrospinning method. To investigate the dependence of the MIS capacitor performance on the residual PVDF and the TiO_2_ phase, the annealing process was optimized at rapid thermal annealing (RTA) temperatures of 300, 450, and 600 °C. Then, a Ti metal layer was deposited by sputtering using a Ti target to fabricate an anatase TiO_2_-based heterostructured MIS capacitor. The electrospinning method provides a new way to tailor the electrical properties of MIS capacitors by controlling various parameters, in order to form films with the desired structure according to the target application. This paper highlights that electrospinning can be performed without vacuum equipment, making the process very low cost and applicable to semiconductor applications. However, further research is required for nanoscale processing of semiconductors.

## 2. Experimental Section

First, the solution was prepared to fabricate the TiO_2_ dielectric layer, followed by the electrospinning process

### 2.1. Materials and Solution Preparation

Titanium(IV) oxide (TiO_2_, anatase form, JUNSEI, Tokyo, Japan) and PVDF (Mw~534,000, Sigma Aldrich, Seoul, Republic of Korea), acting as binder, were mixed in a 94:6 (4.7 g of TiO_2_, 0.3 g of PVDF) mass ratio; then, N-methyl-2-pyrrolidone (NMP, 99.7%, JKC, Cheonan, Republic of Korea), used as solvent, was added at about 7 mL to obtain the appropriate viscosity. The solution was stirred at 800 rpm for 24 h (magnetic stirrer, WISD, Seoul, Republic of Korea). Additionally, sonication and stirring were repeated several times in an ultrasonic cleaner (DAIHAN Scientific, Seoul, Republic of Korea) to disperse the TiO_2_ phase.

### 2.2. Electrospinning

In general, the electrospinning process is used to produce nano–microscale fibers; however, in order to prepare a relatively uniform film and increase the adhesion between the silicon wafer and the dielectric layer, PVDF-based electrospraying was performed with the concentration of the electrospinning solution reduced to ~3–4 wt.%. In this experiment, PVDF was used for the electrospinning polymer solution. PVDF has various advantages such as high production stability and abundant green resources, and the PVDF phase can be controlled by electrospinning, which makes it a promising electrode material. PVDF is also used as an anode binder in lithium-ion batteries and is often used to improve the structural stability, so it was also used in this experiment [25,26,27]. Electrospinning was performed using a single nozzle (nozzle adaptor, NanoNC, Seoul, Republic of Korea) and a constant flow rate of 2 mL/h using a syringe pump (Fusion 100-X precision dosing two-channel syringe pump, Chemyx, Stafford, TX, USA). To improve the uniformity of the dielectric layer, a drum-type collector (NNC-DC90H, NanoNC, Seoul, Republic of Korea) was used as a spin coater, and the wafer was spun in place at 200 rpm. The distance between the drum collector and the syringe tip was set to 17 cm, and a high voltage of 8–10 kV was applied. Finally, a 23-gauge plastic nozzle (inner diameter: 0.33 mm, outer diameter: 0.63 mm, NanoNC, Seoul, Republic of Korea) was used to prevent clogging at the tip when TiO_2_ particles flowed through the syringe at a constant rate.

### 2.3. Fabrication of MIS Capacitor

**Figure 1:**
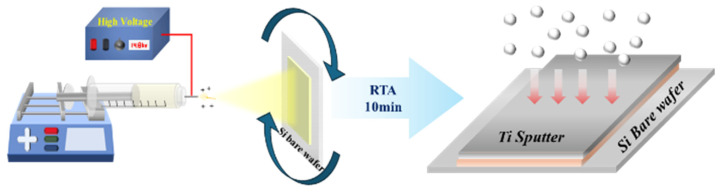
Schematic illustration of the fabrication of the electrospun TiO_2_ dielectric layer and Ti-sputtered metal layer.

In this experiment, rapid thermal process (RTP) annealing (KVR-2000, Korea Vacuum Tech, Goyang, Republic of Korea) was used to anneal the TiO_2_ dielectric layer at RTA temperatures of 300, 450, and 600 °C to observe changes in crystallinity and electrical properties with the annealing temperature. In the RTP, the N_2_ gas was flowed at 500 sccm under vacuum conditions. Afterward, the sputtering device (RF magnetron sputtering system KVS-2004, Korea Vacuum Tech) was used to deposit the metal layer. Using a Ti target, the Ar gas was flowed inside the chamber at 18 sccm under high vacuum conditions. After adjusting the pressure inside the chamber to 2.2 × 10^−2^ torr, the Ar plasma was formed with an RF power of 200 mW to deposit Ti for 120 min; finally, the MIS Ti–TiO_2_–Si heterostructure was successfully fabricated (Figure 1).

### 2.4. Analysis

Scanning electron microscopy (SEM), atomic force microscopy (AFM), and Raman spectroscopy were used to evaluate the surface state, roughness, adhesion force, and composition of the electrospun MIS capacitor (see Table S1). X-ray diffraction (XRD) analysis was performed to determine crystalline phase changes with the annealing temperature. Finally, a probe station was used to evaluate the electrical properties.

Raman spectroscopy measurements employed a XperRAM35V (NanoBase, Seoul, Republic of Korea) instrument with a 532 nm laser and a 1800-lpmm grating. AFM experiments were carried out with a NX-10 (ParkSystems, Suwon, Republic of Korea) instrument; a Tap300Al-G cantilever (force constant 40 N/m, resonance frequency 300 kHz) was applied for non-contact mode experiments, whereas a PPP-CONTSCR cantilever (force constant 40 N/m, resonance frequency 300 kHz) was used for measuring force–distance curves. Field-emission SEM (FE-SEM, SIGMA300, Carl Zeiss, Oberkochen, Germany) was used to analyse the deposition conditions, thickness, and interlayer separation of the film. Finally, the crystal structure of TiO_2_ was characterized by XRD using Cu Kα radiation (New D8-Advance, Bruker-AXS, Karlsruhe, Germany).

## 3. Results and Discussion

**Figure 2:**
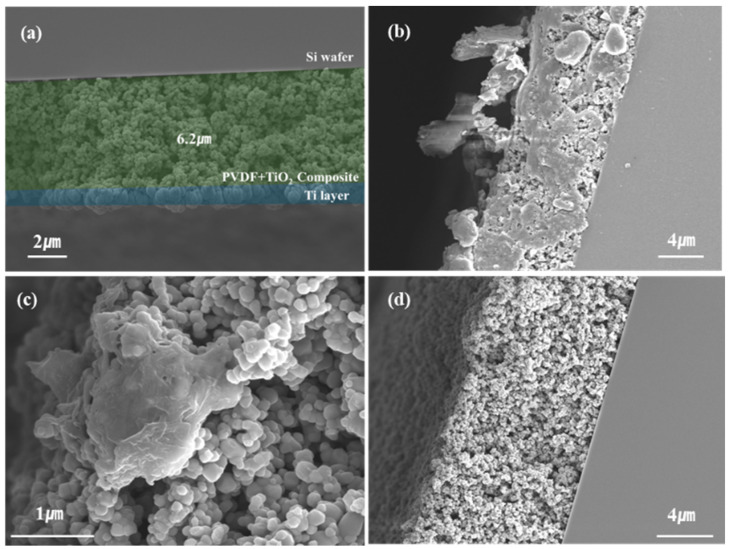
Cross-section of FE-SEM images of (**a**) electrospun porous TiO_2_ dielectric films in the Ti–TiO_2_–Si heterostructure for MIS capacitors annealed at 600 °C, as well as electrospun TiO_2_ dielectric films annealed at RTA temperatures of (**b**) 300 °C, (**c**) 450 °C, and (**a**,**d**) 600 °C.

Figure 2 shows SEM images of electrospun TiO_2_ samples annealed at RTA temperatures of 300, 450, and 600 °C. The above samples were electrospun at 2 mL/h for a total of 5 min, and the dielectric layer had a thickness range of ~6–10 μm. A thinner film could be produced by reducing the electrospinning process time. Figure 2a shows the final electrospun MIS structure obtained in this study. Figure 2a is a cross-sectional SEM image of a hetero-structured MIS capacitor with a TiO_2_ dielectric layer deposited by electrospinning and a Ti metal layer deposited by sputtering after 600 °C RTA heat treatment. A Ti metal layer was deposited on the electrospun TiO_2_ using a sputter, as shown in more detail in Figure S1. Figure 2b shows the electrospun MIS structure of the sample annealed at an RTA temperature of 300 °C for 10 min. A large amount of residual PVDF can be seen in the figure. Moreover, Figure 2c shows the sample annealed at an RTA temperature of 450 °C for 10 min; a small amount of PVDF was still present, although much smaller than that of the 300 °C-annealed sample. Finally, Figure 2d shows the sample annealed at an RTA temperature of 600 °C for 10 min: most PVDF was volatilized during the annealing process, and no residual PVDF was visible under the optics. The amount of PVDF in the dielectric layer affects the leakage current. In this experiment, PVDF was used to act as a binder for TiO_2_. Although PVDF can improve the insulation properties, it exhibits polarization due to the hysteresis effect in the electric field, which leads to energy loss [28]. Therefore, the amount of residual PVDF will affect the leakage current. To confirm the morphology and roughness of electrospun TiO_2_ as a function of temperature, the AFM data are shown in detail in Figure S2.

**Figure 3:**
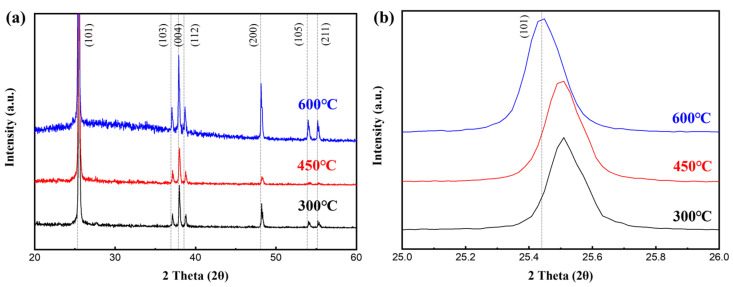
XRD patterns of electrospun TiO_2_ dielectric layers annealed at RTA temperatures of 300, 450, and 600 °C; 2θ ranges: (**a**) 20°–65°, (**b**) 25°–26°.

The XRD patterns in Figure 3 show that the crystallinity of the TiO_2_ thin films improved with increasing annealing temperature. The XRD analysis was performed before the Ti electrode was deposited. TiO_2_ anatase phase peaks corresponding to (101), (103), (004), (112), (200), (105), and (211) orientations were observed at 2θ = 25.7°, 37.1°, 38°, 38.7°, 48°, 54°, and 55°, respectively [29]. More details of XRD patterns of the TiO_2_ electrospun thin films are shown in Figure S3. The intensity of the (101) peak, representative of anatase TiO_2_, increased with the RTA temperature. The anatase peak intensities of the 450 and 600 °C annealed samples increased by ~9.3% and 25.2%, respectively, compared to that of the sample annealed at 300 °C. Kang et al. reported that, among the two main TiO_2_ phases, rutile showed a worse leakage current than anatase. It was also reported that the leakage current decreased as the proportion of anatase phase increased, denoting better electrical properties [30]. Therefore, the anatase peak ratio was expected to increase as the annealing temperature increases from 300 to 450 and 600 °C, reflecting better leakage current properties. As shown in Figure 3b, the peaks shifted toward lower 2θ values as the annealing temperature increased. This was because, when the material was annealed, the atoms underwent thermal expansion; this caused the lattice constant to increase, with a corresponding shift of the XRD peaks toward lower angles [31].

**Figure 4:**
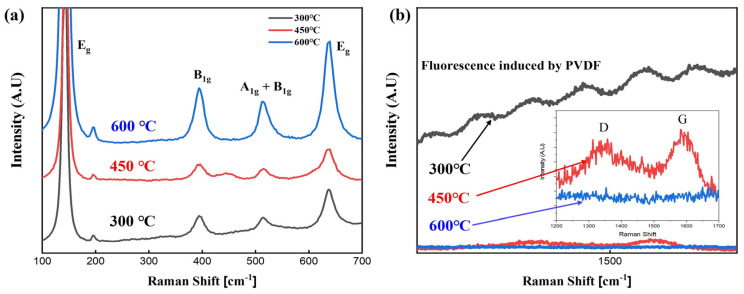
Raman data of electrospun TiO_2_ dielectric layer annealed at RTA temperatures of 300, 450, and 600°. (**a**) Comparison of TiO_2_ peaks; (**b**) enlargement of the organic material region.

Raman spectroscopy can be used to qualitatively evaluate the percentage of residual PVDF in the TiO_2_/PVDF composite thin film and the crystallinity of TiO_2_ particles. As shown in Figure 4 and Figure S4, TiO_2_ anatase peaks (144, 394, 514, 634 cm^−1^) were observed for all samples at 300, 450, and 600 °C, and the peak intensity tended to increase as the annealing temperature increased. In addition, the fluorescence caused by PVDF tended to decrease with increasing annealing temperature. This matches the trend of the SEM images, which indicates that the material composition ratio and thin film properties of the TiO_2_/PVDF composite can be controlled by adjusting the annealing temperature conditions.

**Figure 5:**
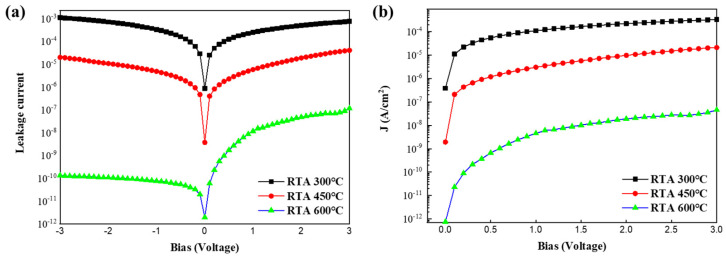
I–V characteristics of Ti–TiO_2_/PVDF composite/Si MIS devices. (**a**) Current–voltage curve in voltage range of −3 to 3 V; (**b**) current density–voltage curve in voltage range of 0 to 3 V.

The current–voltage characteristics were measured for MIS devices consisting of the Ti–TiO_2_/PVDF composite and Si. The applied voltage range was −3 to 3 V. Figure 5a shows the leakage current and Figure 5b is the leakage current density of each sample. The asymmetric I–V relationship shown in Figure 5a is commonly observed in high-k dielectric materials. The asymmetry curve is correlated with the dielectric TiO_2_ thickness [32]. The charge conduction under positive bias is believed to be dominated by the silicon/oxide interface, while that under negative bias is controlled by the metal/oxide interface [13]. As the annealing temperature of the samples increased, the leakage current density at Vg = 0 V decreased to 0.88 μA, 3.7 nA, and 2.0 pA for the 300, 450, and 600 °C samples in Figure 5b, respectively. This is consistent with the XRD and Raman spectroscopy results discussed above. The physical origin of this decrease in leakage current is the N_2_ gas that flows during annealing. This is because, depending on the annealing temperature, N_2_ is incorporated into the TiO_2_ film, which helps to densify it and reduce the bulk and interfacial defect densities [33,34]. In particular, for the same thickness of the 600 °C sample, the results of this work show better leakage current characteristics than those reported in other studies [8,31]. These leakage current characteristics are related to the amount of residual PVDF, variation in the anatase phase, and N_2_ incorporation as a function of heat treatment temperature.

## 4. Conclusions

In this study, a dielectric thin film was fabricated on a Si wafer using a TiO_2_/PVDF electrospinning solution with NMP as solvent. Generally, to fabricate MIS capacitors, each layer is deposited by CVD, ALD, and other techniques; however, in this study we used an electrospinning deposition method, which has never been reported before. Although electrospinning is commonly used to produce polymer nanofibers, in this study the dielectric layer was deposited by electrospraying. Compared to other deposition methods, electrospinning provides various advantages, such as a significantly shorter processing time, lower operating costs, and no requirement for vacuum equipment. In addition, the desired film thickness can be achieved by adjusting the processing time and, in the case of MIS capacitors, the optimal dielectric constant can be obtained by controlling the additive in the electrospinning solution.

Raman spectroscopy and XRD measurements showed that the fabricated thin films exhibited different leakage current characteristics depending on the annealing temperature, indicating a reduction in the residual PVDF amount and activation of the TiO_2_ anatase phase. Among the fabricated thin films, the sample annealed at 600 °C showed the best leakage current (2.0 pA). This demonstrates the superior performance of TiO_2_/PVDF composites as dielectric thin films. However, further research is needed to adjust the surface roughness and develop a process to form thin films with nanoscale thickness. As future work, we will also study the behavior of capacitance and cell potential of electrospun TiO_2_ MIS.

To develop the next generation of capacitor devices, not only TiO_2_ but also high-k materials such as HfO_2_ can be added to the electrospinning solution, or the dielectric layer can be fabricated by mixing two or more materials with different k values, in order to match the dielectric constant to the application. The electrospinning-based coating is also one of the best methods for producing flexible films, as polymer solutions are utilized in the era of smaller scales and flexible electronic devices. Although electrospinning is still a process at the hundreds of nano- to multi-micro-scale, it has many advantages, and further research is required for its application in the semiconductor field.

## Data Availability

The data presented in this study are available on request from the corresponding author.

## Supplementary Materials

The following supporting information can be downloaded at https://www.mdpi.com/article/10.3390/mi15101231/s1. Figure S1: SEM image of sputtered Ti on the electrospun TiO_2_ dielectric layer. Figure S2: The 5 × 5 µm^2^ area AFM 3D topography (a) 300 ℃, (b) 450 °C, and (c) 600 ℃ annealed samples. Figure S3: XRD data of an electrospun TiO_2_ dielectric layer annealed at RTA temperatures of 300, 450, and 600 °C. (a) shows 2theta from 20° to 65°, (b) shows 2theta from 36° to 40°, (c) shows 2theta from 68.5° to 70.5°, and (d) shows 2theta from 25° to 26°. Figure S4: Raman data of an electrospun TiO_2_ dielectric layer annealed at RTA temperatures of 300, 450, and 600 degrees. Table S1: Bond strength and surface roughness (R_a_) as annealing temperature varies.
