# Supplementary material for: Fabrication of Electrospun Porous TiO2 Dielectric Film in a Ti–TiO2–Si Heterostructure for Metal–Insulator–Semiconductor Capacitors
Source: Micromachines (Basel). 2024 Sep 30;15(10):1231. doi: 10.3390/mi15101231 (PMC11509767; doi:10.3390/mi15101231)
Supplement: Supplementary file 1 [file micromachines-15-01231-s001.zip › micromachines-3228352-supplementary.pdf]

micromachines

MDPI

Supplementary Information: Fabrication of Electrospun Porous TiO2 Dielectric Film in a Ti–TiO2–Si Heterostructure for Metal–Insulator–Semiconductor Capacitors

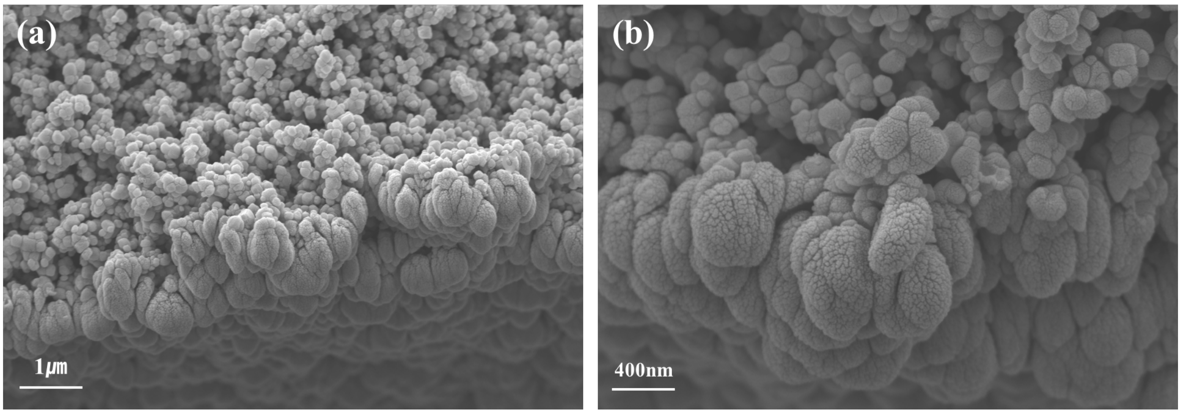

Figure S1. SEM Image of sputtered Ti on the electrospun TiO2 dielectric layer.

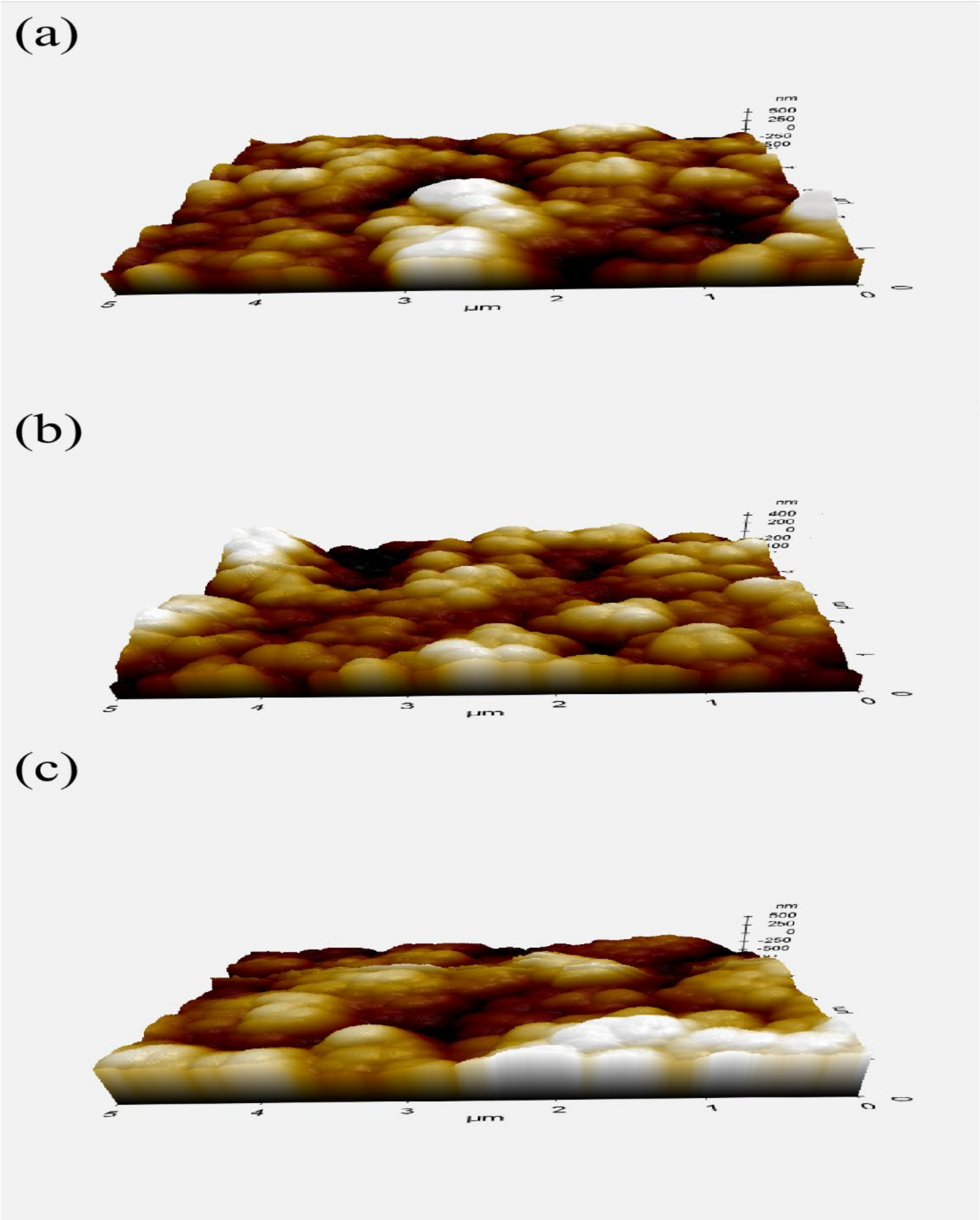

Figure S2. 5 x 5 μm ² Area AFM 3D topography (a) 300°C, (b) 450°C, (c) 600°C annealed sample.

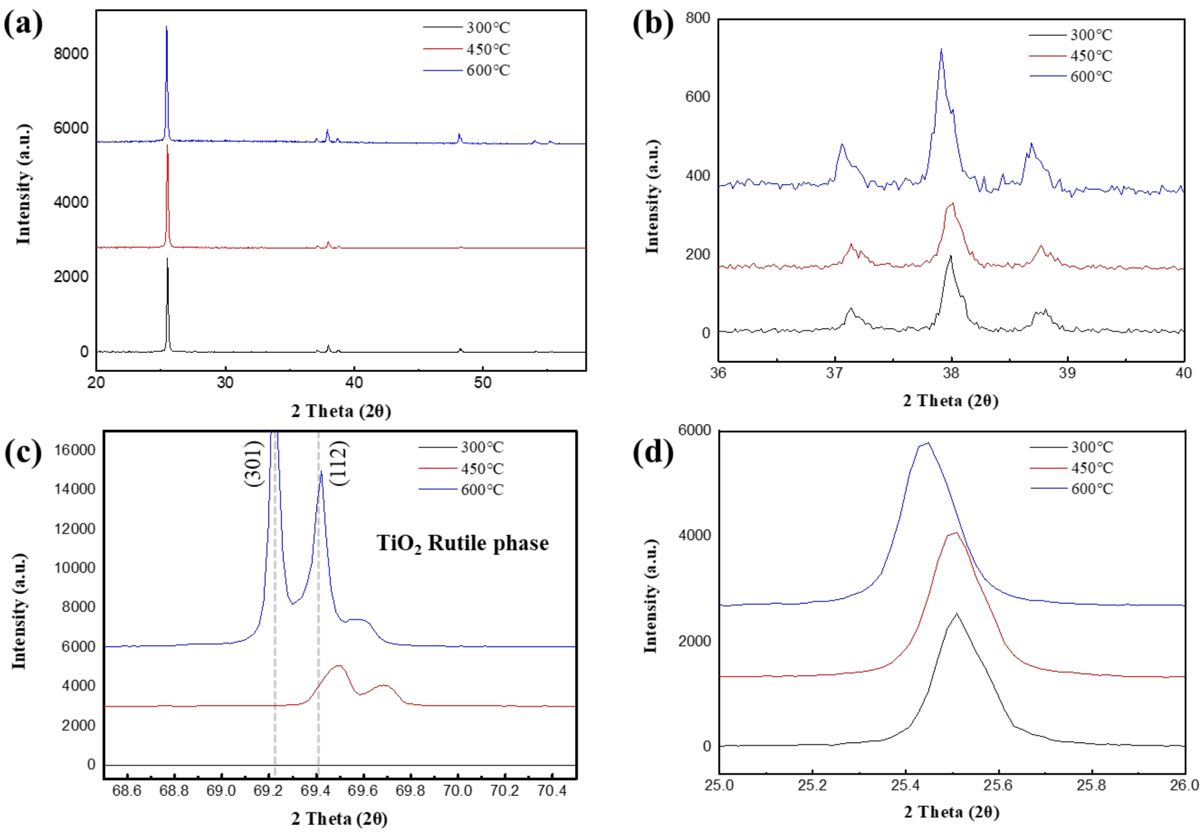

Figure S3. XRD data of an electrospun TiO_2 dielectric layer annealed at RTA 300,450,600℃. (a) shows 2theta from 20˚ to 65˚, (b) shows 2theta from 36˚ to 40˚, (c) shows 2theta from 68.5˚ to 70.5˚ and (d) shows 2theta from 25˚ to 26˚.

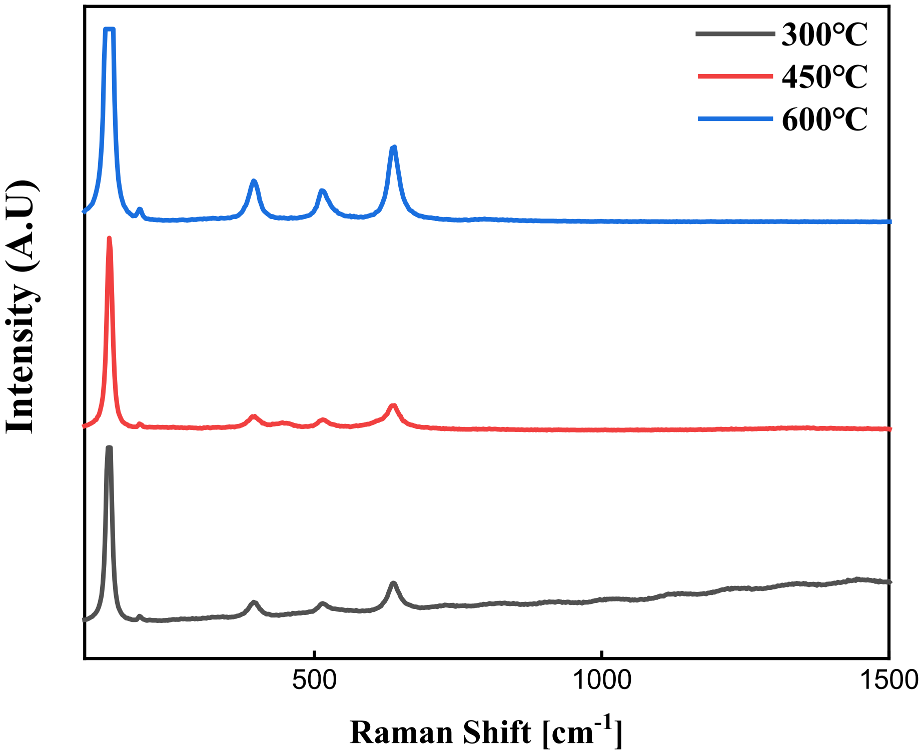

Figure S4. Raman data of an electrospun TiO_2 dielectric layer annealed at RTA 300,450,600 degrees.

AFM Analysis

For AFM, NX-10 of ParkSystems was used, and Tap300Al-G (Force Constant of 40 N/m, Resonance Frequency of 300 kHz) Cantilever was applied for non-contact mode, and PPP-CONTSCR Cantilever (Force Constant of 40 N/m, Resonance Frequency of 300 kHz) was used for Force - Distance Curve measurement. The mapping size is 5 x 5 μm^2

Through AFM analysis, the surface roughness and bond strength of the thin film were measured. For surface roughness, non-contact mode was applied to measure without damaging the soft sample, and for bond strength, contact mode was applied to extract F-D curve. As shown in Table 1, bond strength and surface roughness tended to increase as the annealing temperature increased. the bond strength was calculated as follows.

$$W = \frac{F}{1.5\pi R_a}, R_a = \frac{RR_s}{R+R_s} \ , \ R_s = \frac{(h^2+r^2)}{2h}$$

$W = Bonding\ Strength$
$F = Adhesion\ Focre$
$R = Radius\ of\ Probe$
$h = Average\ surface\ grain\ height$
$r = radius\ of\ Surface\ grain\ Curvature$

Table S1. Bond Strength and Surface Roughness (R_a) as annealing temperature varies.

Annealing temperature [°C]	Bond Strength [J/m^2]	R_a [nm]
300	0.166	109
450	0.171	118
600	0.310	158
